# Modulation of NaCl-induced osmotic, cytogenetic, oxidative and anatomic damages by coronatine treatment in onion (*Allium cepa* L.)

**DOI:** 10.1038/s41598-023-28849-w

**Published:** 2023-01-28

**Authors:** Dilek Çavuşoğlu

**Affiliations:** Department of Plant and Animal Production, Plant Protection Program, Atabey Vocational High School, Isparta University of Applied Sciences, Isparta, Turkey

**Keywords:** Plant physiology, Cell biology

## Abstract

Coronatine (COR), a bacterial phytotoxin produced by *Pseudomonas syringae*, plays important roles in many plant growth processes. Onion bulbs were divided four groups to investigate the effects of COR against sodium chloride (NaCl) stress exposure in *Allium cepa* L. root tips. While control group bulbs were soaked in tap water medium, treatment group bulbs were grown in 0.15 M NaCl, 0.01 µM COR and 0.01 µM COR + 0.15 M NaCl medium, respectively. NaCl stress seriously inhibited the germination, root lenght, root number and fresh weight of the bulbs. It significantly decreased the mitotic index (MI), whereas dramatically increased the micronucleus (MN) frequency and chromosomal aberrations (CAs). Moreover, in order to determine the level of lipid peroxidation occurring in the cell membrane, malondialdehyde (MDA) content was measured and it was determined that it was at the highest level in the group germinated in NaCl medium alone. Similarly, it was revealed that the superoxide dismutase (SOD), catalase (CAT) and free proline contents in the group germinated in NaCl medium alone were higher than the other groups. On the other hand, NaCl stress caused significant injuries such as epidermis/cortex cell damage, MN formation in epidermis/cortex cells, flattened cells nuclei, unclear vascular tissue, cortex cell wall thickening*,* accumulation of certain chemical compounds in cortex cells and necrotic areas in the anatomical structure of bulb roots. However, exogenous COR application significantly alleviated the negative effects of NaCl stress on bulb germination and growth, antioxidant defense system, cytogenetic and anatomical structure. Thus, it has been proven that COR can be used as a protective agent against the harmful effects of NaCl on onion.

## Introduction

The genus *Allium*, one of the largest monocot plant genera, has about 920 species^1^. *Allium cepa* L. is commonly known as onion. It has been included in the *Amaryllidaceae* family and *Allioideae* subfamily in recent taxonomic classifications^2^. A biennial herb, *Allium cepa* L. has additional roots, yellowish leaves, and bulbs made from concentric and enlarged fleshy leaf bases. The outer leaf base forms the protective layer and is dry, thin and of various colours. As the onion develops, the inner leaf bases thicken. A mature onion can be long, spherical or oval in shape, and its size varies by variety^3^. *Allium cepa* L. is an important cultivated plant consumed as a vegetable. Although this plant is mostly consumed as food, its antidiabetic, antioxidant and antimicrobial effects are also widely used. This species contains various vitamins, minerals, sulfur amino acids, flavonoids, phytosterols and saponins^4^.

One of the most important environmental factors limiting the normal growth and development of plants is salinity^5,6^. Plants are generally extremely sensitive to soil salinity during germination and early growth^7,8^. Today, approximately 23% of the total irrigated agricultural land is affected by high salinity due to the artificial irrigation methods used in modern agriculture^9^. Various morphological, anatomical, physiological, cytogenetic and biochemical responses may occur in plants exposed to salt stress^10–13^. In addition, it causes osmotic and oxidative stresses by increasing formation of reactive oxygen species (ROS) including free radicals, hydrogen peroxide and singlet oxygen^14–16^. ROS induce a series of responses such as membrane degradation, lipid peroxidation, protein denaturation, antioxidant enzyme inactivation and DNA mutation^16,17^. Plants can cope with salinity-induced osmotic and oxidative stress by operating various mechanisms, including ion partitioning, upregulation of antioxidant activities and osmoregulation^18–20^. Moreover, they can provide osmotic compatibility by increasing the biosynthesis of compatible solutes such as soluble sugars, proline and proteins^5,21^. To mitigate and repair the detriment of salt stress, plants also have a wide range of the antioxidant defense system includes antioxidant enzymes (such as catalase, peroxidase and superoxide dismutase) and non-enzymatic antioxidants (such as salicylate, glutathione, ascorbate, carotenoids and tocopherols). Therefore, it is extremely important to increase the activities of antioxidant enzymes and the content of non-enzymatic compounds to improve the tolerance of plants to salt stress^7,22–26^.

Phytotoxins are highly toxic substances synthesized by plants and plant pathogens. These substances, which are mostly produced as secondary metabolites in plants, are also called phytochemicals, plant allelochemicals and plant poisons^27–29^. These toxins, which accumulate on plant surfaces and tissues, can be found naturally in roots, bark, leaves, fruits and flowers^28,30,31^. Phytotoxins not only protect plants against various biotic and abiotic stress factors, but also serve as growth enhancers and defense proteins, promoting plant growth and survival^32–34^. Phytotoxins are used in agriculture as plant growth regulators and biochemical agents for plant and cell physiology, as they generally have stimulant effects on plants at low concentrations^35–37^.

Coronatine (COR), a non-host-specific phytotoxin, is produced by several members of the *Pseudomonas syringae* group of pathovars^38–40^. The structure of COR is an amide of coronafacic acid and coronamic acid; it is a methyl cyclopropyl amino acid derived from isoleucine^41,42^. COR is a new biotic plant growth regulator, structurally and functionally similar to jasmonates (JAs) such as jasmonic acid, jasmonoyl-isoleucine and methyl jasmonate^43–45^. Although the activities of COR and JAs are similar, they are not the same^46^. Studies have shown that COR are biologically more effective than JAs in the production of secondary metabolites such as protease inhibitors, glyceollin, sakuranetin, momilactone A, alkaloid, nicotine, volatile substances and taxol^47–51^. It has been determined that the physiological effects of COR, a bacterial phytotoxin, vary depending on the plant species^46^ and tissue type^52^. It plays important roles in many plant growth processes such as seed germination^53^, seedling growth^54^, ethylene emission^55^, auxin synthesis^56^, anthocyanin production^57^, alkaloid accumulation^51^, tendril coiling^58^, chlorosis in leaf tissues^59^, leaf senescence^52^, photosynthesis^60^, stomatal opening^61^, reactive oxygen species (ROS) production^62^, lipid peroxidation^63^ and antioxidant enzyme activity^64^. Moreover, the micro-doses of exogenously applied COR can increase tolerance or resistance of plants to different abiotic stresses such as salinity^65^, osmotic^66^, drought^62,67^, heat^68^ and chilling^64,69^.

*Allium* test is a fast, inexpensive and sensitive method. This test associated two aims: toxicity and mutagenicity^70^. Mutagenicity is connected with the chromosome breakdown rate and toxicity is evaluated by observing inhibition root growth. The *Allium* test sensitivity is on par with test systems using algae or human lymphocytes. The results of many tests using a variety of biological organisms yielded results similar to those of the *Allium* test. This has made the mentioned test a reliable scanning test^71,72^. Moreover, the *Allium* test has been proven to be an effective test for genetic monitoring of environmental pollutants in joint studies conducted by WHO (World Health Organization), USEPA (US Environmental Protection Agency) and UNEP (United Nations Environment Programme)^73^.

Although there are very few studies, made in some plant species, on the effects of exogenous COR on the physiological and biochemical parameters examined in the current study under salt stress, unfortunately, there is no study on the effects on cytogenetic parameters and root anatomical structure. On the other hand, the effects of COR on all parameters examined in onion have never been studied and therefore the role of COR in salt stress tolerance of onions has been reported for the first time. Thus, the current study focused on improving the negative effects of osmotic and oxidative stresses induced by NaCl on germination and seedling growth and reducing genotoxicity and anatomical damage in onion plant with exogenous COR application.

## Materials and methods

### Test plant, salt and applied chemical dose

*Allium cepa* L. bulbs, commonly known as onion, were used as plant material. The concentration used in the experiments of COR purchased from Sigma-Aldrich Company, United Kingdom was 0.01 µM. Salt (NaCl) concentration used was 0.15 M. These levels were designated by a preliminary study. Experimental research on plant samples, including the supply of plant material, complies with institutional, national and international guidelines and legislation.

### Germination and growth procedure

Germination experiments were carried out in the dark in an incubator with a temperature of 20 °C and no light. Onion bulbs of approximately the same size and healthy appearance were selected and divided into four main groups (Table 1).

**Table 1:** Main groups and administration doses.

Main groups	Administration doses
Group I/control	Tap water
Group II	0.15 M NaCl
Group III	0.01 µM COR
Group IV	0.01 µM COR + 0.15 M NaCl

Twenty bulbs from each test group were placed in plastic tubs and left to germinate in the incubator for seven days. Bulbs reaching a root length of 10 mm were considered germinated. On the last day of the experiment, the number of root and germination percentages of the bulbs were determined, and root lengths (mm) and fresh weight (g seedling^−1^) were measured. For statistical evaluation, all experiments were arranged in triplicate.

### Procedure for determining cytogenetic differences

For cytogenetic examinations, 1–1.5 cm long pieces of bulb roots germinated for a few days were cut with a razor blade and kept in saturated paradichlorobenzene for 4 h. Then, these fractions were fixed in 3/1 ethanol-acetic acid solution and stored in 70% ethanol. These pieces were hydrolyzed in 1 N HCl for 17 min at 60 °C, stained with Feulgen for 1–1.5 h, crushed on a slide in a drop of 45% acetic acid and covered with a coverslip^74^. One day later, balm was applied around these coverslips and made into permanent preparations. Mitosis stages and mitosis abnormalities seen in root tip meristem cells of onion bulbs were photographed at 100× magnification with a digital camera mounted on a light microscope. MI was calculated by counting a minimum of 30,000 cells (10,000 cells for per slides) from each of the 4 main groups and CAs as % of 2000 dividing cells (for per slide) counted.

### Procedure for determining antioxidant capacity

A quantity (0.2 g) of germinated bulb roots were weighed and homogenized with 5 mL of 50 mM chilled sodium phosphate buffer (pH 7.8). The homogenates were centrifuged at 10,000 rpm for 20 min and the supernatant obtained was used for internal analysis of SOD and CAT antioxidant enzymes.

To determine the SOD content in the root tip cells of germinated onion bulbs, 1.5 mL 0.05 M sodium phosphate buffer (pH 7.8), 0.3 mL 130 mM methionine, 0.3 mL 750 μM nitroblue tetrazolium chloride, 0.3 mL 0.1 mM EDTA–Na_2_, 0.3 mL 20 μM riboflavin, 0.01 mL supernatant, 0.01 mL 4% polyvinylpyrrolidone and 0.28 mL deionized water were added in a test tube and a reaction mixture was prepared. Then, the reaction was started by keeping the tube containing this mixture under two pieces of 15 W fluorescent lamps for 10 min and the reaction was terminated by keeping it in the dark for 10 minutes^75^. SOD activity was expressed as U mg^−1^ FW by measuring absorbance at 560 nm^76^.

To determine the CAT content in root tip meristem cells of germinated bulbs, a 2.8 mL reaction mixture was prepared containing 0.3 mL of 0.1 M H_2_O_2_, 1.5 mL of 200 mM sodium phosphate buffer and 1.0 mL of deionized water. The reaction was started by adding 0.2 mL of supernatant to this mixture and the decrease in 240 nm absorbance as a result of H_2_O_2_ consumption was measured with a UV–Vis spectrophotometer at 25 °C and the CAT activity was determined as OD240nm min g^−1^ FW^77^.

### Procedure for determining cell membrane injury

A quantity (0.5 g) of the fresh roots of the germinated onion bulbs were taken, homogenized with 5% TCA solution in a homogenizer and centrifuged for 15 min at 12,000 rpm at 24 °C. Then, in a 20% TCA solution, 0.5% TBARS and the supernatant were transferred to a different equal volume test tube and allowed to boil at 96 °C for 25 min. At the end of the period, these tubes were placed in an ice bath and centrifuged at 10,000 rpm for 5 min. The absorbance was measured at 532 nm, the MDA content was calculated using the extinction coefficient of 155 M^−1^ cm^−1^ and expressed as µmol g^−1^ FW^78^.

### Procedure for determining free proline accumulation

A quantity (0.5 g) of frozen root tips were weighed and homogenized in 10 mL of 3% aqueous sulfosalicylic acid solution and the homogenates were filtered into a test tube with filter paper. Then, 2 mL of acid-ninhydrin and 2 mL of glacial acetic acid were added to 2 mL of filtrate and incubated at 100 °C for 1 h. This mixture was mixed with 4 mL of toluene and the chromophore containing toluene was separated from the hydrated phase. It was read spectrophotometrically at 520 nm absorbance using toluene as blank. The free proline content was calculated according to a standard curve and expressed as µmol g^−1^ FW^79^.

### Procedure for determining root anatomical differences

Root tips of 1 cm long were cut from germinated onion bulbs to observe the anatomical damage and changes caused by NaCl and COR applications. Root tips were washed with distilled water to remove residues on the surface of the onion roots. Then, cross-sections were taken from the root tips with a sharp razor blade and after staining with 0.5% methylene blue for 2 min, the stained samples of each group were examined with a research microscope at 500× magnification.

### Evaluation of the obtained data

All data obtained from this study were analyzed with the help of SPSS statistics V 23.0 (2015) package program and expressed as mean values by taking their standard deviations (± SD). Statistical analysis of mean values was determined by Duncan's multiple range test (DMRT) and p < 0.05 was considered highly significant.

### Ethical approval and informed consent

Not applicable: This study does not directly involve humans or animals. Plant collection permits were not required because seed samples are commercial cultivars which can be purchased and no species are endangered or threatened.

## Results and discussion

### Effect of COR on the physiological parameters

**Figure 1:**
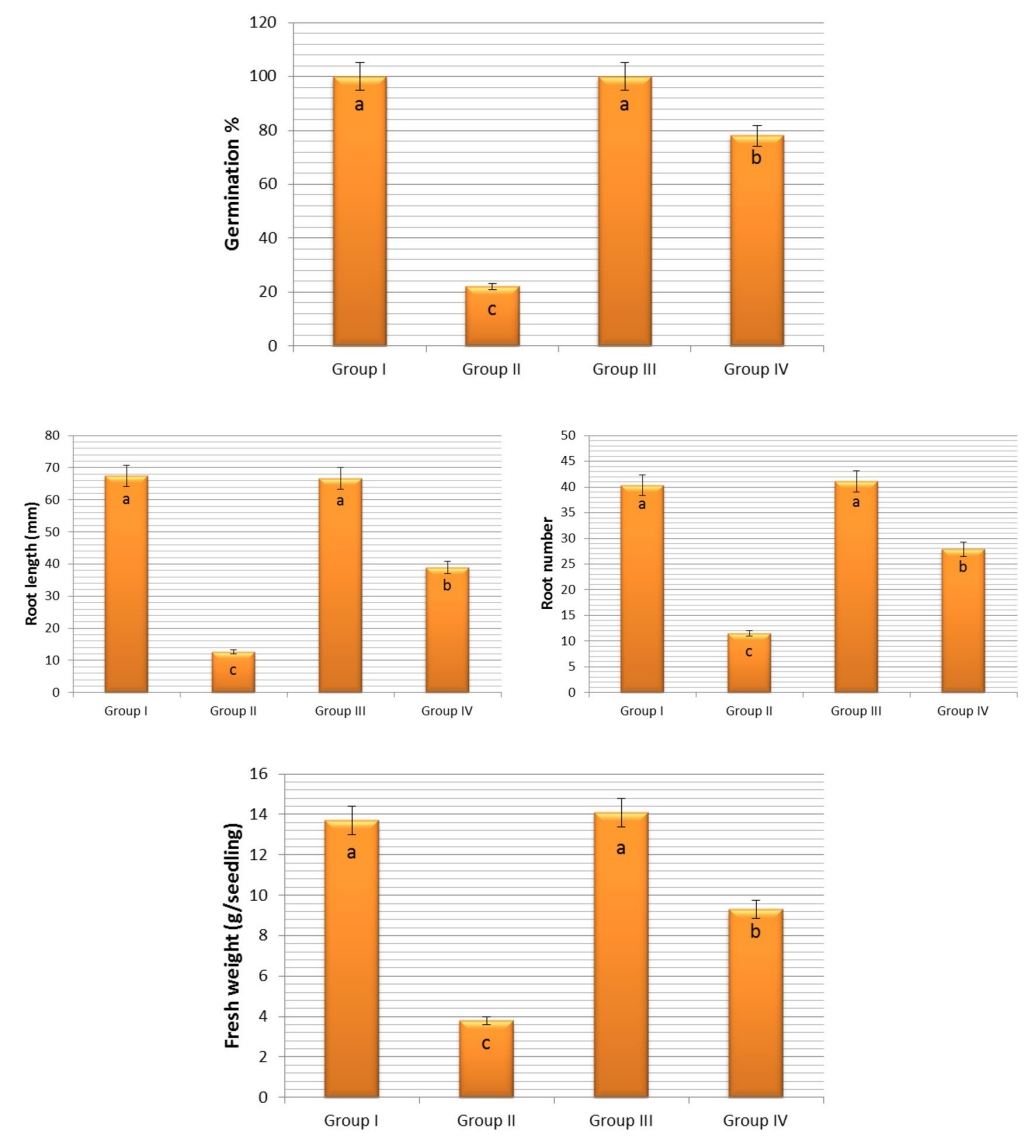
Effect of COR on some physiological parameters of *Allium cepa* L. Group I (control) was treated with tap water; Group II was treated with 0.15 M NaCl; Group III was treated with 0.01 µM COR; Group IV was treated with 0.01 µM COR + 0.15 M NaCl. The error bars indicate the standard deviation (± SD).

Exogenous COR treatment was ineffective on the germination and growth of onion bulbs under normal conditions. That is, the germination percentage, root length, root number and fresh weight of Group III bulbs germinated in medium containing COR alone showed statistically the same values as the bulbs of the control group (Group I) germinated in tap water medium (Fig. 1). It has been found that COR decreases the seed germination^53^ and seedling growth^46,57,80^ at high concentrations, while it stimulates^63^ or does not affect^54,62,65^ at low concentrations. Both the results of available research showing that COR applied in micro doses does not affect the germination and growth of onion bulbs in stress-free environments and the results of above-mentioned researches have proven that this chemical has different effects depending on the plant species, application dose and pretreatment form.

**Figure 2:**
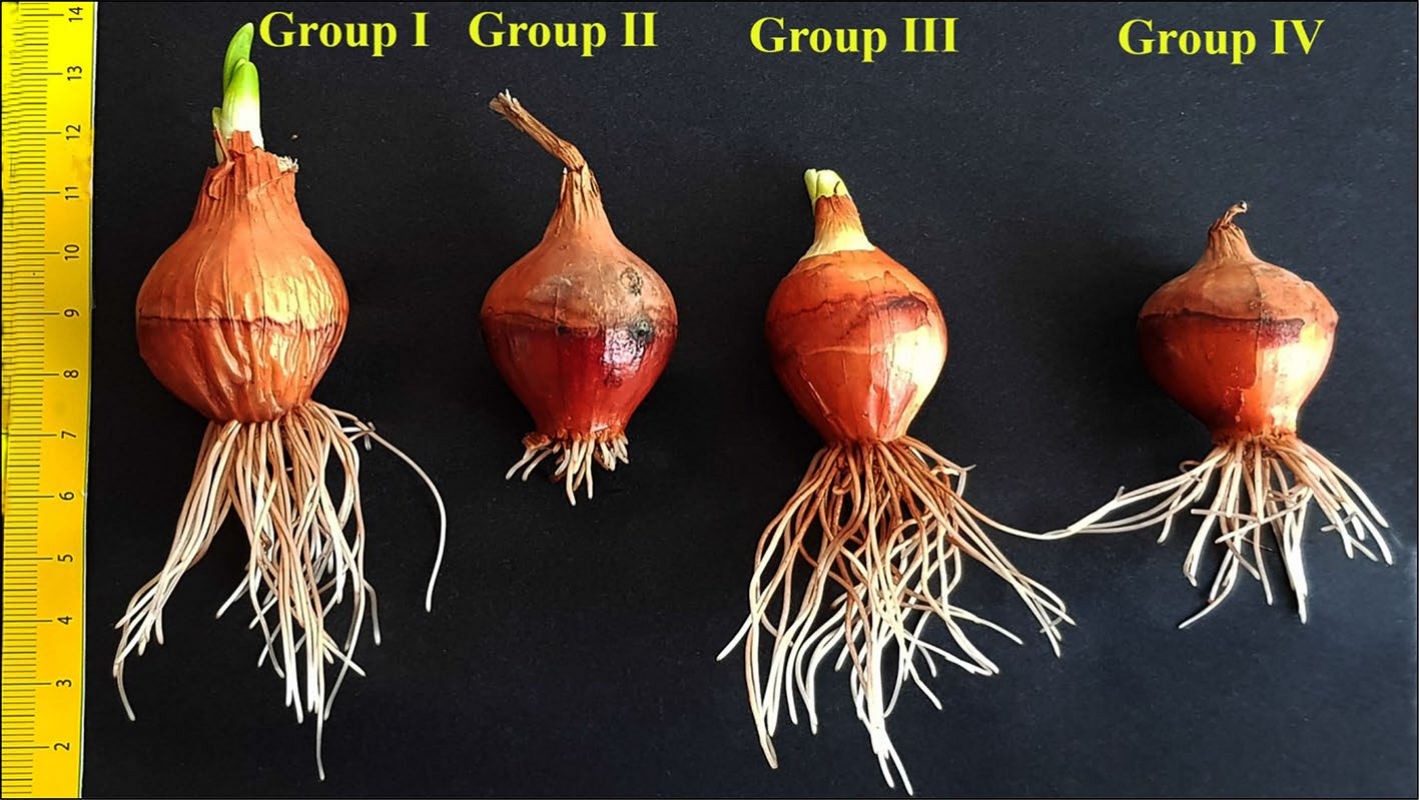
The germination situations at the end of seventh day of *Allium cepa* L. bulbs. Group I was treated with tap water, Group II was treated with 0.15 M NaCl, Group III was treated with 0.01 µM COR, Group IV was treated with 0.01 µM COR + 0.15 M NaCl.

**Figure 3:**
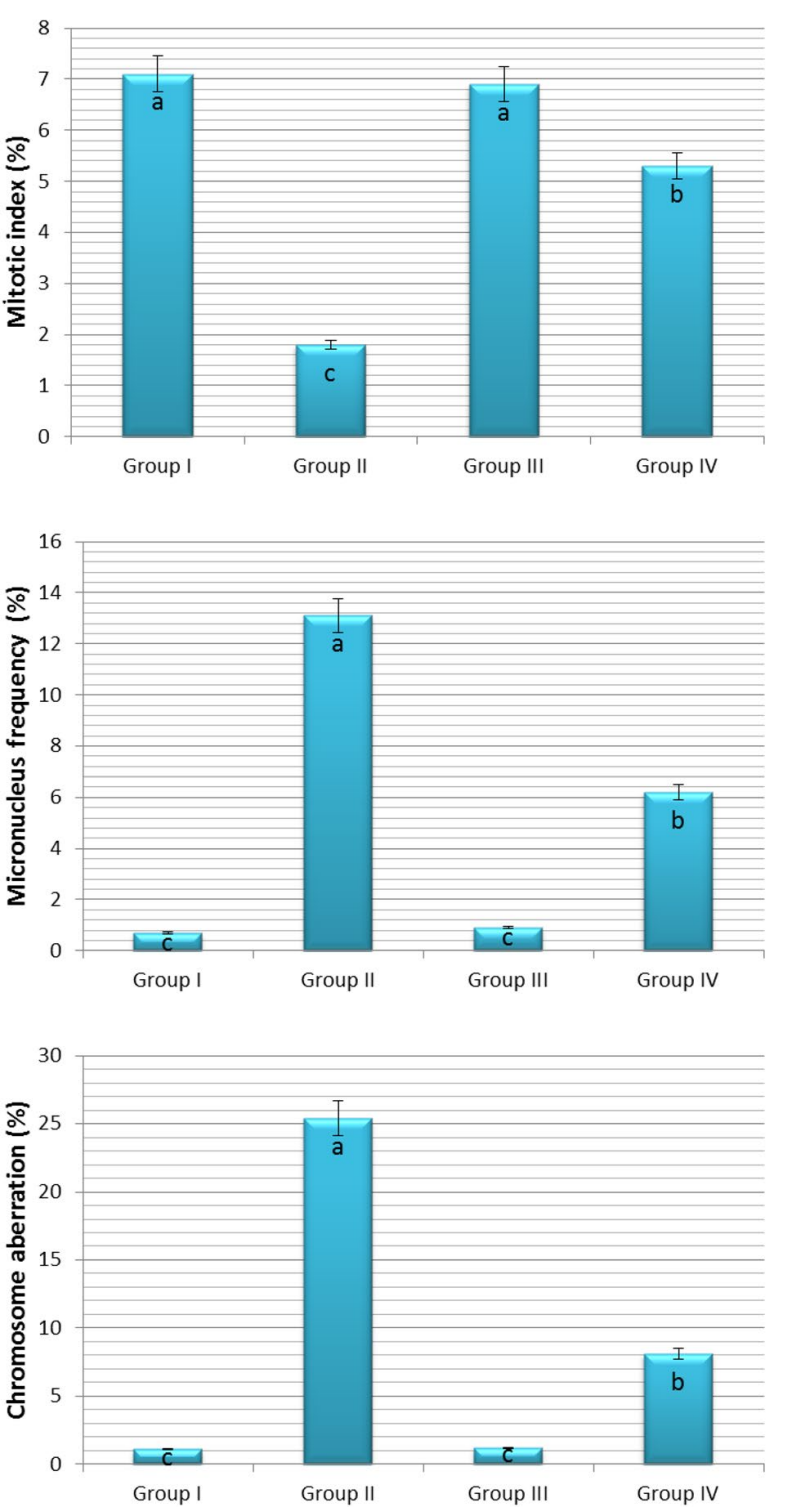
Effect of COR on some cytogenetic parameters of *Allium cepa* L. Group I (control) was treated with tap water; Group II was treated with 0.15 M NaCl; Group III was treated with 0.01 µM COR; Group IV was treated with 0.01 µM COR + 0.15 M NaCl. The error bars indicate the standard deviation (± SD).

**Figure 4:**
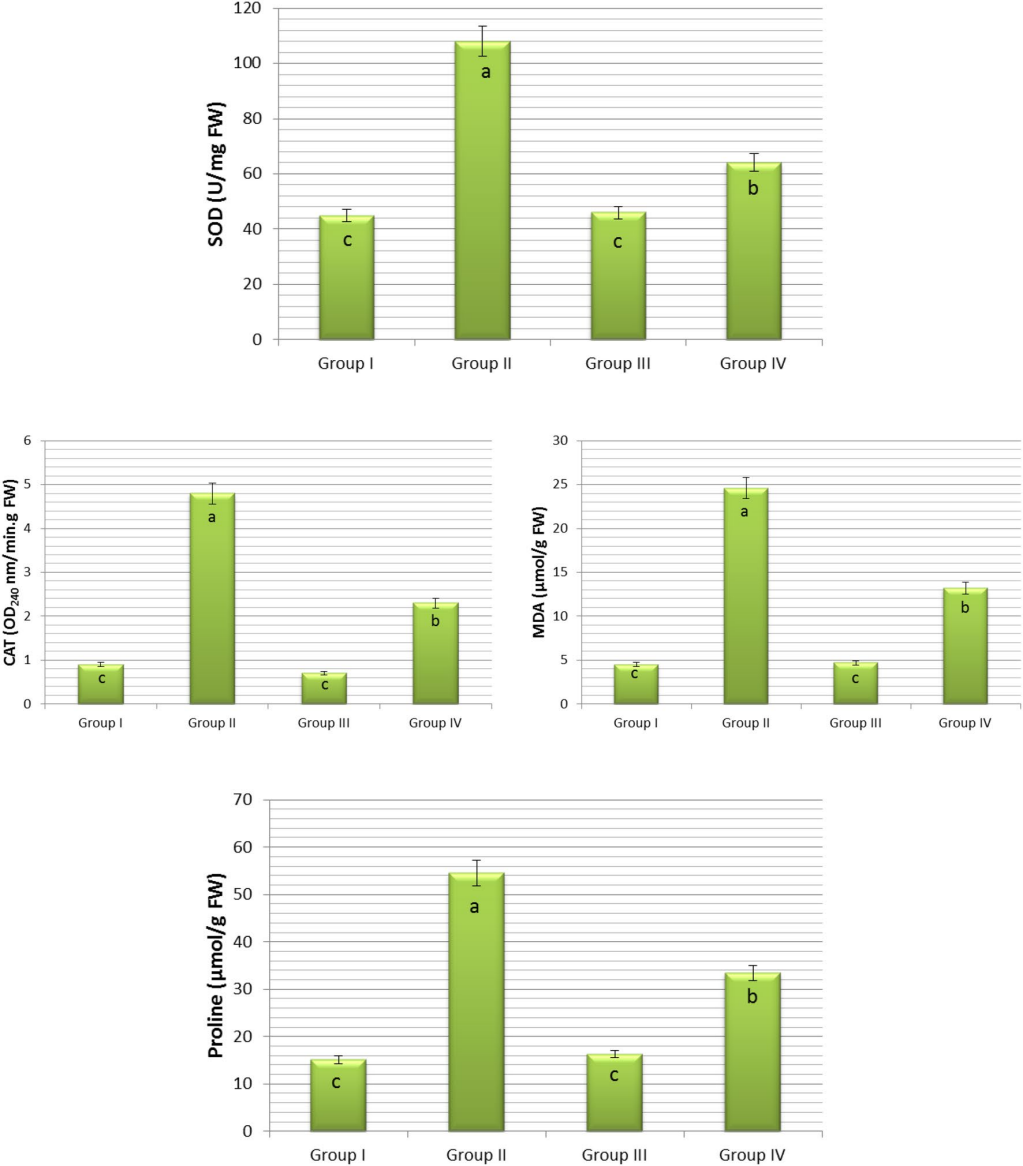
Effect of COR on some biochemical parameters of *Allium cepa* L. Group I (control) was treated with tap water; Group II was treated with 0.15 M NaCl; Group III was treated with 0.01 µM COR; Group IV was treated with 0.01 µM COR + 0.15 M NaCl. The error bars indicate the standard deviation (± SD).

It has been known for a long time that salinity causes adverse effects even on the growth and development of halophytes^81,82^. Salt stress has a negative effect on germination^12,83,84^ and seedling growth^85–87^ and has replicated its negative effect on all physiological parameters examined of onion in this study (Fig. 1). While the germination percentage of Group I bulbs, known as control group, germinated in tap water medium at the end of the experiment (seventh day) was 100 ± 0.0%, this rate was 22 ± 2.4% in Group II bulbs germinated in 0.15 M NaCl medium and thus salt stress reduced the germination of bulbs by 78%. In addition, root length, root number and fresh weight of Group I (control) bulbs grown in tap water medium were 67.4 ± 2.5 mm, 40.3 ± 2.3 and 13.7 ± 1.5 g, respectively. These parameters were determined as 12.6 ± 1.1 mm, 11.5 ± 1.4 and 3.8 ± 0.7 g in Group II bulbs grown in 0.15 M NaCl medium (Figs. 1, 2). These values were statistically significant (p < 0.05). Salt stress can exert its negative effect on germination by inhibiting water uptake of bulbs, by reducting growth promotors (cytokinins and gibberellins) in bulbs and by increasing the growth inhibitors (abscisic acid, ABA) in bulbs^88–91^. Due to the high osmotic pressure of the 0.15 M NaCl medium, the fresh weight and water content of the bulbs may have decreased due to the inability of the roots to receive sufficient water (Fig. 1). In addition, this NaCl concentration may have caused a reduction in the root length and root number of bulbs as it inhibited the mitotic activity in root tip meristematic cells (Fig. 3). Addition of COR to 0.15 M NaCl medium significantly increased the germination of onion bulbs. At this salt level, the germination of COR treated Group IV bulbs reached 78 ± 2.8%. Exogenous COR application also showed a positive effect on the root length, root number and fresh weight parameters. The root length, root number and fresh weight of Group II bulbs grown in 0.15 M NaCl medium were 12.6 ± 1.1 mm, 11.5 ± 1.4 and 3.8 ± 0.3 g, respectively. These parameters were 38.9 ± 1.8 mm, 27.9 ± 1.9 and 9.3 ± 1.1 g in COR-treated Group IV bulbs grown at this salt level (Figs. 1, 2). These values were statistically significant (p < 0.05). Few studies have been conducted about the role of exogenous COR on the fresh weight of seedlings grown under salt stress, but no studies about its effects on the germination percentage, root elongation and root number have been conducted. Only, Xie et al.^63,65^ reported that 0.01 µM COR treatment enhanced the fresh weight of cotton seedlings grown under salt-stressed conditions; and these results were agreement with findings of the present study. COR may have attenuated the NaCl-induced inhibition on the germination and seedling growth by increasing the water uptake of roots (Fig. 1), by stimulating the mitotic activity of root tip meristematic cells (Fig. 3), by reducing lipid peroxidation in root tip meristem cells (Fig. 4) or by regulating the proline content and antioxidant enzyme activities of root cells (Fig. 4).

### Effect of COR on the cytogenetic parameters

It has been reported that the exogenous application of various growth-regulating agents during germination and seedling growth under normal conditions causes cell disruptions, mitotic disorders and chromosomal abnormalities^87,92,93^. The cytogenetic results of this study are very important as there are no available reports on the effects of COR on mitotic index (MI) micronucleus (MN) frequency and chromosome aberrations (CAs) in root meristem cells of seedlings grown in both normal and saline conditions. Figure 3 shows the effects of exogenous COR administration on MI, MN frequency and CAs in root meristem cells of *Allium cepa* L. bulbs. The MI, MN frequency and CAs in roots of the control group (Group I) bulbs germinated in tap water medium were 7.1 ± 1.0%, 0.7 ± 0.7% and CAs 1.1 ± 0.3%, respectively. These parameters were 6.9 ± 0.9%, 0.9 ± 0.8% and CAs 1.2 ± 0.5% in roots of Group III bulbs germinated in medium containing COR alone. That is, exogenous COR treatment was ineffective on MI, MN frequency and CAs in the root cells of onion bulbs germinated under normal conditions.

The increase or decrease in MI is an important indicator in determining the cytotoxicity level of a chemical^94^. Salt stress has both inhibitory and cytotoxic effects on mitotic activity^95–97^, and it is well known that high salinity inhibits mitotic activity in root tip cells and causes chromosomal abnormalities^98,99^. Salt stress, as expected, seriously reduced the mitotic activity expressed as MI in root tips of the bulbs. The MI (1.8 ± 0.6%) in root tip meristems of Group II bulbs germinated in the media containing 0.15 M NaCl decreased approximately 75% as compared with Group I (control) bulbs (7.1 ± 1.0%) germinated in tap water medium. Moreover, 0.15 M salinity induced a drastic increase in MN frequency and CAs in the roots of bulbs. The MN frequency and CAs in root tips of the control (Group I) bulbs were 0.7 ± 0.7% and 1.1 ± 0.3%, respectively. These parameters were 13.1 ± 1.8% and 25.4.1 ± 2.1% in Group II bulbs at 0.15 M NaCl concentration. In other words, 0.15 M NaCl caused an increase more than 18-fold in MN frequency and 23-fold in CAs according to the control (Group I). In summary, 0.15 M salinity caused a significant decrease in the MI and a significant increase in the MN formation and CAs. However, the addition of COR to the 0.15 M NaCl medium significantly alleviated the adverse effects of salt stress on the MI, MN formation and CAs. MI, MN frequency and CA of root cells of Group II bulbs grown in 0.15 M NaCl medium were 1.8 ± 0.6%, 13.1 ± 1.8% and 25.4 ± 2.1%, respectively. These parameters ​​were 5.3 ± 0.7%, 6.2 ± 1.3% and 8.1 ± 1.4% in Group IV bulbs treated with COR (Fig. 3). These results showed that the damage of sodium chloride stress on mitotic division of *Allium cepa* L. can be repaired by exogenous COR application.

**Figure 5:**
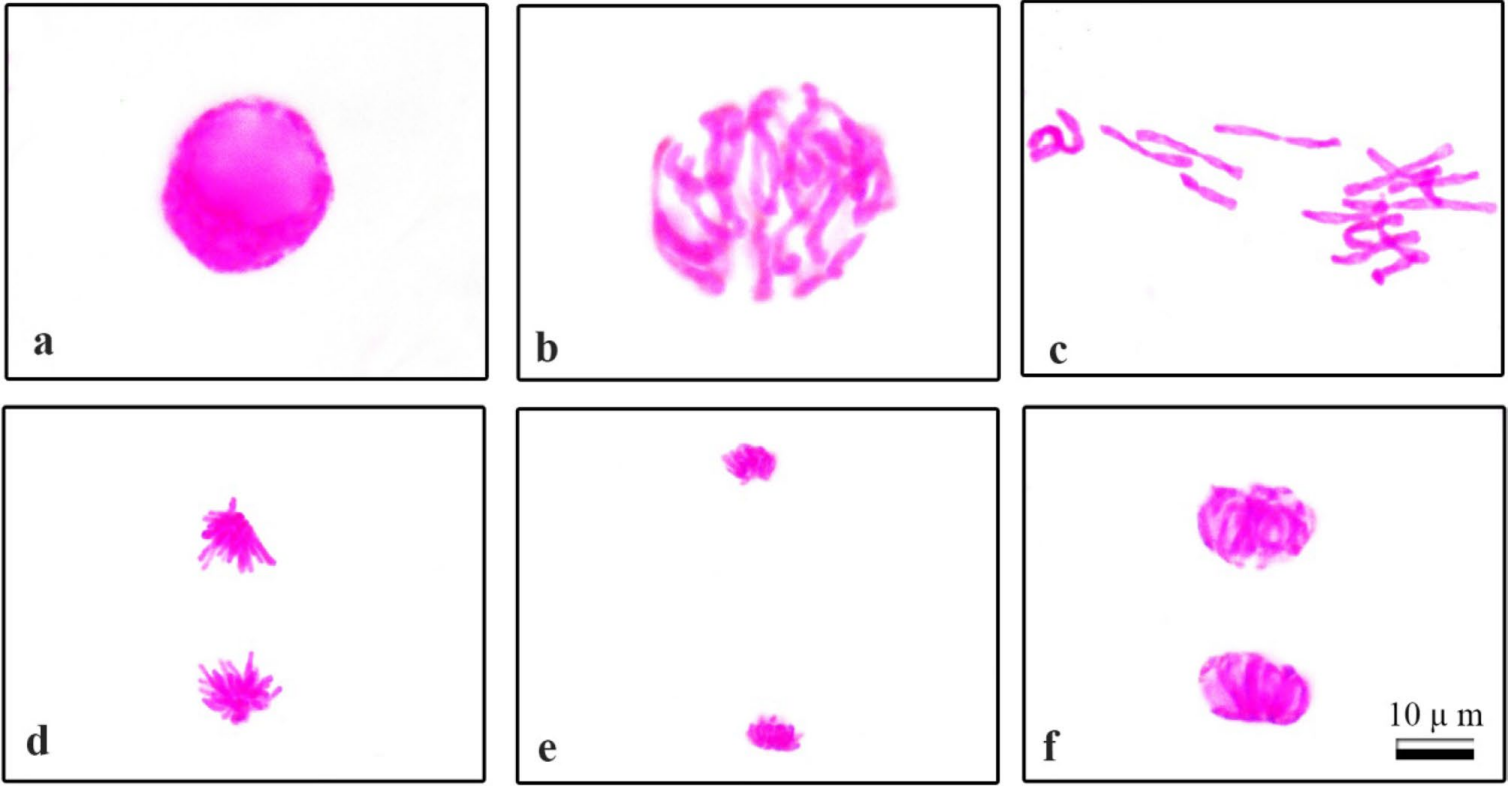
Normal mitosis phases in the roots meristem cells of *Allium cepa* L. grown in tap water and 0.01 µM COR medium (**a**) interphase, (**b**) prophase, (**c**) metaphase, 2n = 16 chromosomes, (**d**) anaphase, (**e**) late anaphase, (**f**) telophase. Scale bar 10 μm.

**Figure 6:**
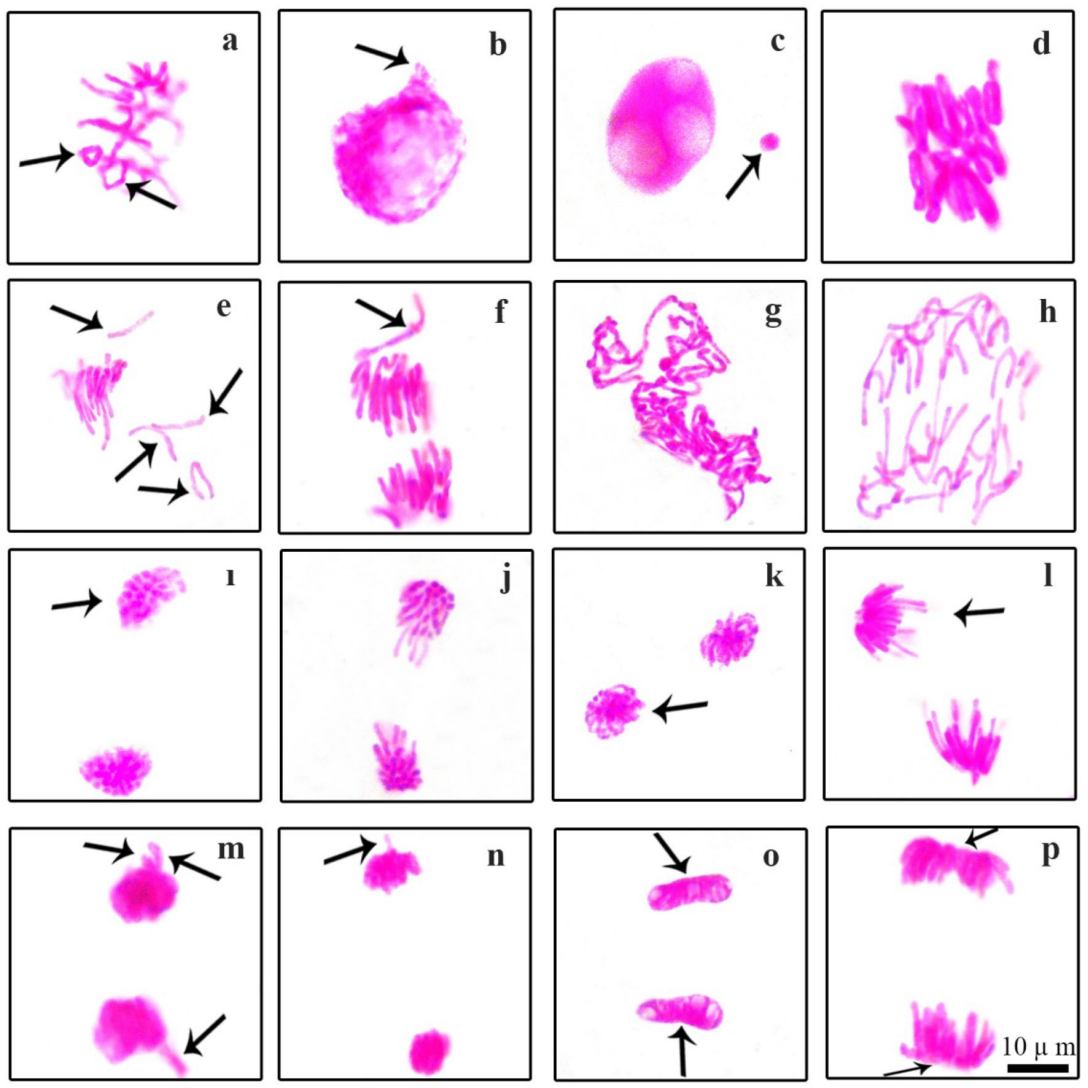
Chromosomal abnormalites in the root meristem cells of *Allium cepa* L. grown in 0.15 M NaCl and 0.01 µM COR + 0.15 M NaCl medium, (**a**) metaphase with chromosome encircleds = arrows, (**b**) nuclear budding** = **arrow, (**c**) trilobulated nucleus with micronucleus = arrow, (**d**) stickiness metaphase, (**e**) metaphase with chromosomal losses = arrows, (**f**) anaphase with chromosomal loss = arrow, (**g**) aberrant prophase, (**h**) aberrant anaphase, (**i**) anaphase with polar slip = arrow, (**j**) chained anaphase, (**k**) telophase with polar slip = arrow, (**l**) anaphase with polar slip = arrow, (**m**) telophase with vagrant chromosomes = arrows, (**n**) anaphase with vagrant chromosome = arrow, (**o**) alignment telophase = arrows, (**p**) alignment anaphase = arrows. Scale bar 10 μm.

Normal and abnormal mitotic stages observed as a result of microscopic examination of meristem cells of bulb roots are shown in Figs. 5 and 6. Common and notable abnormalities were metaphase with chromosome encircleds (Fig. 6a), nuclear budding (Fig. 6b), trilobulated nucleus with micronucleus (Fig. 6c), stickiness metaphase (Fig. 6d), metaphase/anaphase with chromosomal losses (Fig. 6e,f), aberrant prophase/anaphase (Fig. 6g,h), chained telophase/anaphase (Fig. 6i,j), telophase/anaphase with polar slip (Fig. 6i,k,l), telophase/anaphase with vagrant chromosome (Fig. 6m,n) and alignment telophase/anaphase (Fig. 6o,p). Chromosomal or chromosomal breaks that remain in the anaphase stage and cannot combine with both nuclei in the telophase stage lead to the formation of MN^100,101^. Nuclear budding is morphologically uniform to MNs with the exception that they are participate in the nucleus^102^. Formation of MN and formation of cellular budding may be concluded with loss of genetic materials^103^. During the S phase of mitosis, the suppressive effect of a nuclear poison 214 on DNA synthesis causes the formation of lobed nuclei as a nuclear deformation^104^. DNA depolymerization, partial dissolution of nucleoproteins and increased chromosomal contraction and condensation can lead to the formation of stick chromosomes in metaphase. Chromosomal stickiness is an indicator of toxic effects that are irreversible and result in cell death^105^. Chromosomal losses are alteration typically associated to the malfunction of the mitotic spindle^106^. Vagrant chromosome with anaphase/telophase derives from unevenly sized or irregularly shaped nuclei in daughter cells with unequal chromosomes^107^. Spindle disorders lead to anaphase/telophase with fault polarization, which is highly correlated with the incidence of the aforementioned abnormalities other than vacuole nuclei^108^.

### Effect of COR on the biochemical parameters

Reactive oxygen species (ROS) are dangerous cytotoxic molecules, but also act as intermediate signaling molecules to regulate the expression of genes associated with antioxidant defense mechanisms. Plants have antioxidant systems to deal with the damage caused by ROS^15,109,110^ and these systems protect plants from the negative effects of oxidative stress. One of these systems includes antioxidant enzymes such as SOD and CAT^111,112^. Depending on the activity of these enzymes, salt stress tolerance of plants may vary^113,114^. In this study, SOD and CAT contents in roots of the control group (Group I) bulbs germinated in tap water medium were 45 ± 1.8 and 0.9 ± 0.6, respectively. These parameters were 46 ± 2.1 and 0.7 ± 0.2 in roots of Group III bulbs germinated in medium containing COR alone. That is, exogenous COR treatment was ineffective on SOD and CAT activities in the root cells of onion bulbs germinated under normal conditions (Fig. 4). These data obtained indicate that COR did not trigger an additional ROS formation in *Allium cepa* L. roots compared to the control group. These findings were consistent with the findings of researchers who showed that low concentrations of COR did not affect SOD and CAT activity in the leaves and roots of cotton^63,65^, tobacco^62^, chickpea^66^ and maize^54^ grown under normal conditions. On the other hand, NaCl exposure triggered a drastic increase in SOD (108 ± 3.7) and CAT (4.8 ± 1.5) levels in the roots of Group II bulbs. Really, SOD and CAT contents of NaCl-treated root cells approximately risen up to 2.4 and 5.3 folds of their own control (Group I) levels (SOD 45 ± 1.8; CAT 0.9 ± 0.6), respectively (Fig. 4). Parallel results were obtained from studies with plant species such as *Polygonum equisetiforme*^115^, *Astragalus gombiformis*^116^, *Mentha aquatica*^117^, *Mentha pulegium*^118^ and *Chrysanthemum morifolium*^119^. Significant increases in SOD and CAT levels in Group II were reliable signs of ROS formation caused by NaCl. Moreover, increases in MN frequencies, CAs (Fig. 3) and MDA levels (Fig. 4) are an important indicator of NaCl-induced oxidative stress. Oxidative stress causes adverse effects on cell membranes, nucleic acids and other important components of cells. Stimulation of antioxidant enzyme activity can help protect the plant from oxidative damage^120^. CAT and SOD enzymes are enzymatic scavengers of ROS in plants^121^. Of these, SOD converts the superoxide radical to molecular oxygen and H_2_O_2_^122^. CAT catalyzes the degradation of H_2_O_2_ to H_2_O and O_2_^17,123^ thereby increasing membrane stability^124^. Nevertheless, COR addition to NaCl solution contributed to the suppression of oxidative stress. SOD (64 ± 2.6) and CAT (2.3 ± 0.8) contents of Group IV treated with COR were significantly lower than (SOD 108 ± 3.7; CAT 4.8 ± 1.5) Group II in 0.15 M salinity (Fig. 4). The decrease of CAT and SOD enzyme contents in the roots of Group IV bulbs showed that exogenous COR application helped to the fight against ROS in onion plant and increased salt tolerance. However, Xie et al.^63,65^ reported that 0.01 µM exogenous COR application increased SOD and CAT contents in the root of cotton seedlings grown in 150 mM NaCl medium; and these results were not similar to the findings of the present study. These limited research results revealed that the effect of exogenous COR application on SOD and CAT activities may vary depending on the plant species and the degree of exposure to stress.

Oxidative stress caused by salt stress can promote excessive ROS production, which leads to lipid peroxidation^125,126^, which can be determined by measuring the MDA level^127^. In this study, while the MDA content in the roots of Group I bulbs, known as the control group, which germinated in tap water medium, was 4.5 ± 0.7, this parameter was measured as 4.7 ± 0.8 in the roots of Group III bulbs germinated in the medium containing COR alone. In other words, MDA contents were found to be statistically the same in the roots of Group I and Group III bulbs, and exogenous COR did not cause a significant membrane damage in onion root cells (Fig. 4). Similar results were found in cotton^63,65^, chickpea^66^ and maize seedlings^54^ grown in stress-free, that is, normal conditions. On the contrary, NaCl induced a marked increase in MDA (24.6 ± 1.7) content in the roots of Group II bulbs. MDA (24.6 ± 1.7) content of NaCl-treated root cells approximately risen up to 5.5 folds of their own control levels (4.5 ± 0.7). The destructive effect of NaCl-induced oxidative stress on membranes was markedly triggered by increases in MDA content (Fig. 4). These findings were consistent with the findings of researchers who showed that NaCl stress increased the lipid peroxidation in the roots of sweet pepper^128^, tomato^129^, mung bean^130^ and mint^117,131^. On the other hand, COR addition to NaCI solution contributed to the suppression of oxidative stress. Joint application of COR with NaCl lessened by 46% the MDA (13.2 ± 1.2) content of Group IV according to that (24.6 ± 1.7) of Group II in 0.15 M salinity (Fig. 4). Xie et al.^63,65^ determined that 0.01 µM exogenous COR application decreased MDA content in the root of cotton seedlings grown in 150 mM NaCl medium; and these results were agreement with findings of the this study. Haddadi et al*.*^117^ reported that MDA content in tolerant genotypes to salt were lower than the sensitive genotypes. For this reason, the reduction of MDA content by COR in Group IV may be a sign that *Allium cepa* L. provides tolerance to salinity.

Proline is one of the most widely produced osmolytes^132^, which plays an important role in maintaining osmotic potential and turgor in plants exposed to high salinity^133,134^. Moreover, proline also performs the task of protecting cells by stabilizing cellular membranes and proteins during dehydration^135–137^. Although this amino acid is synthesized in plants through glutamate and ornithine, the glutamate pathway is the main source of proline production under osmotic stress^114^. In this study, while the free proline content in the root of Group I (control) bulbs germinated in tap water medium was 15.1 ± 1.7, this parameter was measured as 16.3 ± 2.1 in the root of Group III bulbs germinated in medium containing COR alone, and these two values were statistically similar (Fig. 4). Ceylan et al.^66^ detected that 0.01 µM COR application enhanced the proline content in roots of chickpea plants grown in stress-free conditions; and this conclusion was not consistent with the finding of current research. Whereas, NaCl triggered a drastic increase in the free proline level (54.5 ± 3.9) in the roots of Group II bulbs. The free proline content (54.5 ± 3.9) of NaCl-treated root cells approximately risen up to 3.6 folds of their own control (15.1 ± 1.7) level (Fig. 4). Proline is known to accumulate under saline conditions^115,117,138,139^. However, it is not known whether proline accumulation occurs as a result of the stress effect or stress tolerance^114^. Although a positive correlation between abiotic stress tolerance and free proline accumulation has been reported^136,140,141^, a negative correlation between proline accumulation and salt tolerance has also been reported^137,142,143^. As in this study, a positive correlation was found between MDA and proline accumulation^115^. This shows that proline effectively participates in scavenging the produced ROS and thus protects the cells from oxidative damage^144^. On the other hand, joint application of COR with NaCl decreased the free proline content in the root of Group IV bulbs. The free proline content of Group IV bulbs treated with COR was 33.4 ± 3.0 while this parameter was 54.5 ± 3.9 in Group II bulbs in 0.15 M salinity (Fig. 4). Unfortunately, there are no studies about the effects of exogenous COR on free proline content in roots of plants exposed to NaCl stress. Khedr et al.^145^ determineted that exogenous proline increased the protein content in *Pancratium maritimum* L. under saline conditions. The reduce content of free proline in the roots of Group IV bulbs treated with COR may be due to the generation of new proteins for oxidative stress tolerance.

### Effect of COR on the anatomic parameters

**Figure 7:**
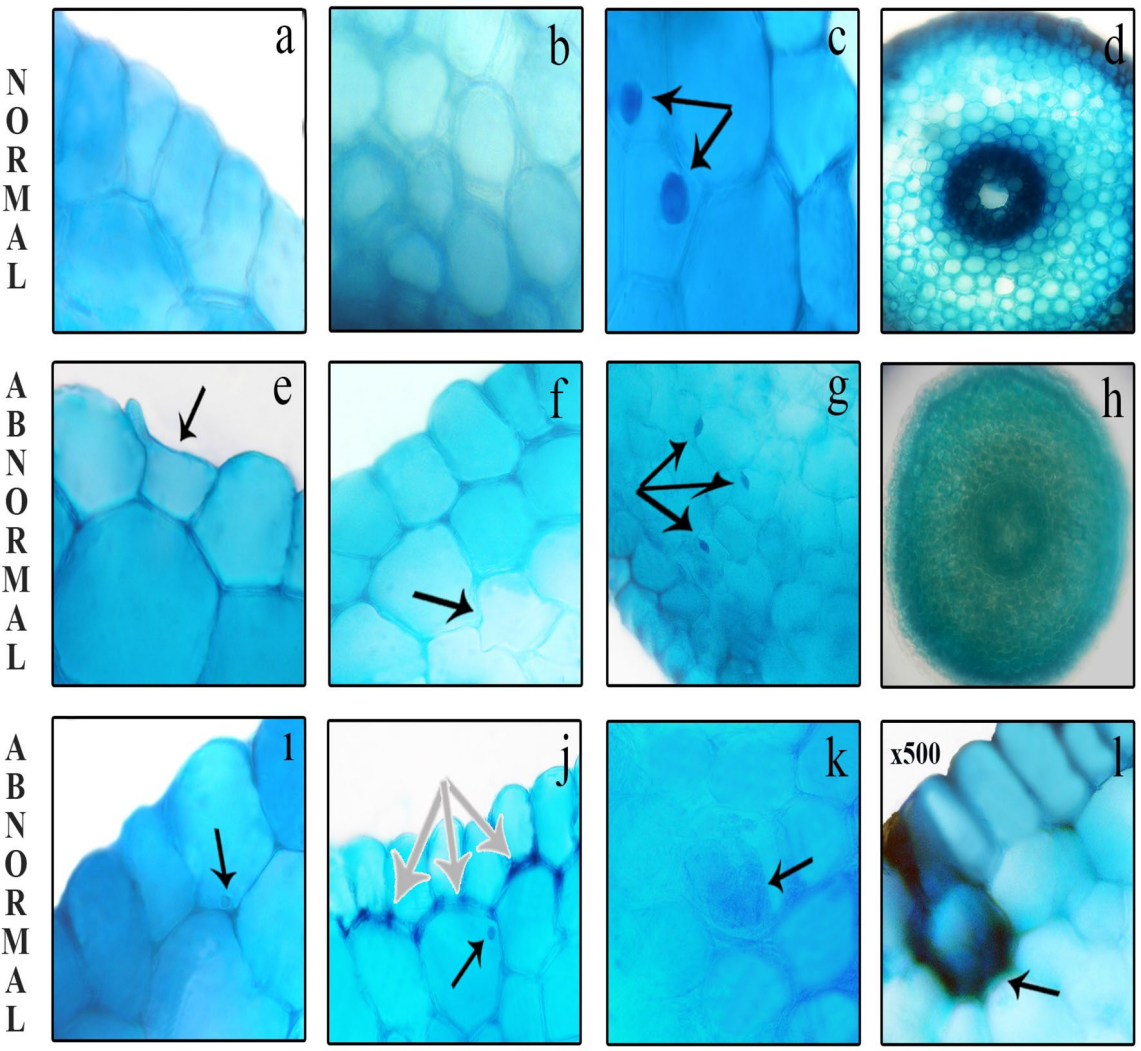
NaCl-induced root anatomical structure damages, (**a**) normal appearance of epidermis cells, (**b**) normal appearance of cortex cells, (**c**) normal appearance of cell nuclei-oval = arrows, (**d**) clear vascular tissue, (**e**) epidermis cell damage = arrow, (**f**) cortex cell damage = arrow, (**g**) flattened cells nuclei = arrows, (**h**) unclear vascular tissue, (**i**) micronucleus formation in epidermis cells = arrow, (**j**) cortex cell wall thickening (white) and micronucleus formation in cortex cells (black), (**k**) accumulation of some chemical compounds in cortex cells = arrow, (**l**) necrotic areas = arrow.

Since the roots are the most vulnerable and first part of the plants, if this organ is exposed to external toxic agents, the most severe damage to the anatomical structure is expected to occur in this part. Anatomic changes observed in root epidermis layer cells are associated with deterioration in the characteristic structure of the cell membrane. Figure 7 and Table 2 show NaCl-induced root anatomical damages of *Allium cepa* bulbs and the protective effect of COR against NaCl-induced structural damages. No damage was detected in the root anatomical structure of the control (Group I) bulbs germinated in tap water medium and Group III bulbs germinated in the medium containing COR alone, as a result of microscopic examinations. In the root anatomical structure of Group II bulbs germinated in 0.15 M salinity determined damages such as epidermis/cortex cell damage (Fig. 7e,f), micronucleus formation in epidermis/cortex cells (Fig. 7i,j), flattened cells nuclei (Fig. 7g), unclear vascular tissue (Fig. 7h), cortex cell wall thickening (Fig. 7j)*,* accumulation of certain chemical compounds in cortex cells (Fig. 7k) and necrotic areas (Fig. 7l). This suggests that these damages are occur as a result of the defense mechanisms of cells and tissues in order to minimize the stress due to the exposure of *Allium cepa* L. to salt stress.

**Table 2:** Alleviation of NaCl-induced root anatomical damages by external COR application.

Groups	ECD	CCD	MNE	MNC	FCN	UVT	CWT	NA	ACC
Group I/control	−	−	−	−	−	−	−	−	−
Group II	+++	+++	++	++	+++	+++	+++	+++	+++
Group III	−	−	−	−	−	−	−	−	−
Group IV	+	+	+	+	+	+	+	+	+

ECD epidermis cell deformation, CCD cortex cell deformation, MNE micronucleus formation in epidermis, MNC micronucleus formation in cortex, FCN fattened cell nucleus, UVT unclearly vascular tissue, CWT cortex cell wall thickening, NA necrotic areas, ACC accumulation of some chemical compounds in cortex cells. (−) no damage, (+) little damage, (++) moderate damage, (+++) severe damage.

As seen in Table 2, the addition of 0.01 µM exogenous COR to 0.15 M NaCl medium reduced to a minor level the severity of these anatomical damages caused by salt stress observed in the root anatomical structure. Epidermis and cortex cell damage may be indication that salt stress causes a toxicity severe enough to disrupt cell wall integrity. Flattened cells nuclei formations can occur not only as a result of rupture of cell membranes, but also as a result of DNA damage due to oxidative stress^146^. When plants are exposed to stress, they develop mechanisms such as reduced substance transport, cortex cell wall thickening and accumulation of some chemical compounds in cells in order to tolerate the harmful effects of chemicals and stress^147^. As a result of these mechanisms, anatomic changes occur in the plant and the harmful effects of chemical agents are reduced. No data have been reported in the literature regarding the effect of COR on the root anatomy of plants grown under both salt stress and normal conditions. Therefore, the root anatomical findings from this study are very important as it is the first to be reported.

## Conclusion

The effects of exogenously applied COR on some physiological, cytogenetic, biochemical and anatomical parameters in the roots of *Allium cepa* L. bulbs germinated in saline (NaCl) conditions have been extensively investigated. There are no available literature data on the effects of COR application under salt stress conditions on all parameters of *Allium cepa* L studied here. Therefore, the results from this study are very important as it is the first time reported in onion. These results showed that the application of COR at appropriate doses can significantly reduce sodium chloride stress on the germination and growth of onion bulbs by regulating osmoregulation, mitotic activity and antioxidant capacity. Moreover, these results may help develop new hypotheses and conceptual tools to increase salt tolerance in plants.

## Data Availability

The datasets used and/or analyzed during the current study are available from the corresponding author on reasonable request.

## References

[1] Herden T, Hanelt P, Friesen N (2016). Phylogeny of *Allium* L. subgenus *Anguinum* (G. Don. ex W.D.J. Koch) N. Friesen (Amaryllidaceae). Mol. Phylogenet. Evol..

[2] Peruzzi L, Carta A, Altinordu F (2017). Chromosome diversity and evolution in Allium (Allioideae, Amaryllidaceae). Plant Biosyst..

[3] Lim TK, Springer (2015). Modified stems, roots, bulbs. Edible Medicinal and Non-Medicinal Plants.

[4] Marrelli M, Amodeo V, Statti G, Conforti F (2019). Biological properties and bioactive components of *Allium cepa* L.: Focus on potential benefits in the treatment of obesity and related comorbidities. Molecules.

[5] Ahanger MA, Agarwal RM (2017). Salinity stress induced alterations in antioxidant metabolism and nitrogen assimilation in wheat (*Triticum aestivum* L.) as influenced by potassium supplementation. Plant Physiol. Biochem..

[6] Lin J, Wang Y, Sun S, Mu C, Yan X (2017). Effects of arbuscular mycorrhizal fungi on the growth, photosynthesis and photosynthetic pigments of *Leymus chinensis* seedlings under salt-alkali stress and nitrogen deposition. Sci. Total Environ..

[7] Li JT, Qiu ZB, Zhang XW, Wang LS (2011). Exogenous hydrogen peroxide can enhance tolerance of wheat seedlings to salt stress. Acta Physiol. Plant..

[8] Wu H (2012). Effects of salinity on metabolic profiles, gene expressions and antioxidant enzymes in halophyte *Suaeda salsa*. J. Plant Growth Regul..

[9] Jouyban Z (2012). The effects of salt stress on plant growth. Tech. J. Eng. Appl. Sci..

[10] Gumi AM, Aliero AA, Shehu K, Danbaba A (2013). Salinity stress: Effects on growth, biochemical parameters and ion homeostasis in *Solanum lycospersicum* L. (cv. Dan eka). Cent. Eur. J. Exp. Biol..

[11] Cavusoglu K, Bilir G (2015). Effects of ascorbic acid on the seed germination, seedling growth and leaf anatomy of barley under salt stress. ARPN J. Agric. Biol. Sci..

[12] Cavusoglu D (2020). Cytogenetical and physiological effects of L-tryptophan in onion (*Allium cepa* L.) exposed to salt stress. Russ. Agric. Sci..

[13] Tabur S, Avcı ZD, Özmen S (2021). Exogenous salicylic acid application against mitodepressive and clastogenic effects induced by salt stress in barley apical meristems. Biologia.

[14] Chen LM, Lin CC, Kao CH (2000). Copper toxicity in the rice seedlings: Changes in antioxidative enzyme activities, H_2_O_2_ level, and cell wall peroxidase activity in roots. Bot. Bull. Acad. Sin..

[15] Neill S, Desikan R, Hancock J (2002). Hydrogen peroxide signalling. Curr. Opin. Plant Biol..

[16] Garg N, Manchanda G (2009). ROS generation in plants: Boon or bane?. Plant Biosyst..

[17] Gill SS, Tuteja N (2010). Reactive oxygen species and antioxidant machinery in abiotic stress tolerance in crop plants. Plant Physiol. Biochem..

[18] Ahmad P (2015). Role of *Trichoderma harzianum* in mitigating NaCl stress in Indian mustard (*Brassica juncea* L) through antioxidative defense system. Front Plant. Sci..

[19] Ahanger MA, Agarwal RM, Tomar NS, Shrivastava M (2015). Potassium induces positive changes in nitrogen metabolism and antioxidant system of oat (*Avena sativa* L. cultivar Kent). J. Plant Interact..

[20] Ahmad P (2016). Nitric oxide mitigates salt stress by regulating levels of osmolytes and antioxidant enzymes in chickpea. Front. Plant Sci..

[21] Ahanger MA, Tittal M, Mir RA, Agarwal RM (2017). Alleviation of water and osmotic stress-induced changes in nitrogen metabolizing enzymes in *Triticum aestivum* L. cultivars by potassium. Protoplasma.

[22] Mittler R (2002). Oxidative stress, antioxidants and stress tolerance. Trends Plant Sci..

[23] Foyer CH, Noctor G (2003). Redox sensing and signalling associated with reactive oxygen in chloroplasts, peroxisomes and mitochondria. Physiol. Plant..

[24] Sairam RK, Tyagi A (2004). Physiology and molecular biology of salinity stress tolerance in plants. Curr. Sci..

[25] Triantaphylides C, Havaux M (2009). Singlet oxygen in plants: Production, detoxification and signaling. Trends Plant Sci..

[26] Bidabadi SS, Mehri H, Ghobadi C, Baninasab B, Afazel M (2013). Morphological, physiological and antioxidant responses of some Iranian grape vine cultivars to methyl jasmonate application. J. Crop Sci. Biotechnol..

[27] Osman AMG, Chittiboyina AG, Khan IA, Morris JG, Potter ME (2013). Plant toxins. Foodborne Infections and Intoxications.

[28] Thakur A, Sharma V, Thakur A (2018). Phytotoxin: A mini review. J. Pharmacogn. Phytochem..

[29] Chen H (2020). An exploration on the toxicity mechanisms of phytotoxins and their potential utilities. Crit. Rev. Environ. Sci. Tech..

[30] Froberg B, Ibrahim D, Furbee RB (2007). Plant poisoning. Emerg. Med. Clin. N. Am..

[31] Patel S, Nag MK, Daharwal S, Singh MR, Singh D (2013). Plant toxins: An overview. Res. J. Pharmacol. Pharmacodyn..

[32] Kobayashi K (2004). Factors affecting phytotoxic activity of allelochemicals in soil. Weed Biol. Manage..

[33] Chadwick DJ, Whelan J (2008). Secondary Metabolites: Their Function and Evolution.

[34] Bucheli TD (2014). Phytotoxins: Environmental Micropollutants of Concern?.

[35] Belz RG, Cedergreen N (2010). Parthenin hormesis in plants depends on growth conditions. Environ. Exp. Bot..

[36] Yamane H, Mander L, Lui HW (2010). Chemical defence and toxins of plants. Comprehensive Natural Products II Chemistry and Biology.

[37] Abbas T, Nadeem MA, Tanveer A, Chauhan BS (2017). Can hormesis of plant released phytotoxins be used to boost and sustain crop production?. Crop Protect..

[38] Bender CL, Alarcon CF, Gross DC (1999). *Pseudomonas syringae* phytotoxins: Mode of action, regulation and biosynthesis by peptide and polypeptide synthetases. Microbiol. Mol. Biol. Rev..

[39] Cintas NA, Koike ST, Bull CT (2002). A new pathovar, *Pseydomonas syringae* pv. alisalensis, proposed for the causal agent of bacterial blight of broccoli and broccoli raab. Plant Dis..

[40] Melotto M, Underwood W, He SY (2008). Role of stomata in plant innate immunity and foliar bacterial disease. Ann. Rev. Phytopathol..

[41] Parry RJ, Mhaskar SV, Lin MT, Walker AE, Mafoti R (1994). Investigations of the biosynthesis of the phytotoxin coronatine. Can. J. Chem..

[42] Greulich F, Yoshihara T, Ichihara A (1995). Coronatine, a bacterial phytotoxin, acts as a stereospecific analog of jasmonate type signals in tomato cells and potato tissues. J. Plant Physiol..

[43] Palmer DA, Bender CL (1995). Ultrastructure of tomato leaf tissue treated with the pseudomonad phytotoxin coronatine and comparison with methyl jasmonate. Mol. Plant-Microbe Interact..

[44] Zhang ZY (2009). Coronatine-induced lateral-root formation in cotton (*Gossypium hirsutum*) seedlings under potassium sufficient and deficient conditions in relation to auxin. J. Plant Nutr. Soil Sci..

[45] Geng XQ, Jin L, Shimada M, Kim MG, Mackey D (2014). The phytotoxin coronatine is a multi functional component of the virulence armament of *Pseudomonas syringae*. Planta.

[46] Uppalapati SR (2005). The phytotoxin coronatine and methyl jasmonate impact multiple phytohormone pathways in tomato. Plant J..

[47] Ichihara A, Toshima H (1998). Recent studies on coronatine. Chem. Regul. Plants..

[48] Tamogami S, Kodama O (2000). Coronatine elicits phytoalexin production in rice leaves (*Oryza sativa* L.) in the same manner as jasmonic acid. Phytochemistry.

[49] Fliegmann J, Schüler G, Boland W, Ebel J, Mithofer A (2003). The role of octadecanoids and functional mimics in soybean defense responses. Biol. Chem..

[50] Lauchli R, Boland W (2003). Indanoyl amino acid conjugates: Tunable elicitors of plant secondary metabolism. Chem. Rec..

[51] Schuler G (2004). Coronalon: A powerful tool in plant stress physiology. FEBS Lett..

[52] Kenyon JS, Turner JG (1990). Physiological changes in *Nicotiana tobacum* leaves during development of chlorosis caused by coronatine. Physiol. Mol. Plant Pathol..

[53] Lin AI (2008). Physiological effects of coronatine on seed germination of upland and lowland rice. Acta Agric..

[54] Wang BQ (2008). Effects of coronatine on growth, gas exchange traits, chlorophyll content, antioxidant enzymes and lipid peroxidation in maize (*Zea mays* L.) seedlings under simulated drought stress. Plant Prod. Sci..

[55] Kenyon JS, Turner JG (1992). The stimulation of ethylene synthesis in *Nicotiana tabacum* leaves by the phytotoxin coronatine. Plant Physiol..

[56] Liu Y (2020). Coronatine inhibits mesocotyl elongation by promoting ethylene production in etiolated maize seedlings. Plant Growth Regul..

[57] Li Y, Huang G, Guo Y, Zhou Y, Duan L (2020). Coronatine enhances stalk bending resistance of maize, thickens the cell wall and decreases the area of the vascular bundles. Agronomy.

[58] Uppalapati SR (2008). Pathogenicity of *Pseudomonas syringae* pv. tomato on tomato seedlings: Phenotypic and gene expression analyses of the virulence function of coronatine. Mol. Plant Microbe. Interact..

[59] Brooks DM, Bender CL, Kunkel BN (2005). The *Pseudomonas syringae* phytotoxin coronatine promotes virulence by overcoming salicylic acid-dependent defenses in *Arabidopsis thaliana*. Mol. Plant Pathol..

[60] Hao L (2013). Coronatine enhances drought tolerance via improving antioxidative capacity to maintaining higher photosynthetic performance in soybean. Plant Sci..

[61] Zhou YL, Irene VV, Robert CS, Corne MJP, Saskia CMVW (2017). Atmospheric CO_2_ alters resistance of *Arabidopsis* to *Pseudomonas syringae* by affecting abscisic acid accumulation and stomatal responsiveness to coronatine. Front. Plant Sci..

[62] Xu J, Zhou Y, Xu Z, Chen Z, Duan L (2020). Combining physiological and metabolomic analysis to unravel the regulations of coronatine alleviating water stress in tobacco (*Nicotiana tabacum* L.). Biomolecules.

[63] Xie ZX, Duan LS, Li ZH, Wang XD, Liu XJ (2015). Dose-dependent effects of coronatine on cotton seedling growth under salt stress. J. Plant Growth Regul..

[64] Wang L (2009). Coronatine enhances chilling tolerance in cucumber (*Cucumis sativus* L.) seedlings by improving the antioxidative defence system. J. Agron. Crop Sci..

[65] Xie ZX (2008). Coronatine alleviates salinity stress in cotton by improving the antioxidative defense system and radical-scavenging activity. J. Plant Physiol..

[66] Ceylan HA, Turkan I, Sekmen AH (2013). Effect of coronatine on antioxidant enzyme response of chickpea roots to combination of PEG-induced osmotic stress and heat stress. J. Plant Growth Regul..

[67] Ai L (2008). Coronatine alleviates polyethylene glycol-induced water stress in two rice (*Oryza sativa* L.) cultivars. J. Agron. Crop Sci..

[68] Zhou Y (2015). Phytotoxin coronatine enhances heat tolerance via maintaining photosynthetic performance in wheat based on electrophoresis and TOF-MS analysis. Sci. Rep..

[69] Qi FG, Li JM, Duan LS, Li ZH (2006). Inductions of coronatine and Meja to low temparature resistance of wheat seedling. Acta Bot. Boreal. Occid. Sin..

[70] Tedesco, S. B. & Laughinghouse, I. V. H. D. Boindicator of genotixicity: The *Allium cepa* test, Environmental Contamination. (ed. Srivastava, J.) 138–156. http://www.intechopen.com/books/environmental-contamination/bioindicator-of-genotoxicitythe-allium-cepa-test. (In Tech, 2012).

[71] Fiskesjo G (1985). The Allium test as a standard in environ-1535 mental monitoring. Hereditas.

[72] Chaparro TR, Botta CM, Pires EC (2010). Biodegradability and toxicity assessment of bleach plant effluents treated anaerobically. Water Sci. Technol..

[73] Grant WF (1999). Higher plant assays for the detection of chromosomal aberrations and gene mutations—a brief historical back ground on their use for screening and monitoring environmental chemicals. Mutat. Res..

[74] Sharma PC, Gupta PK (1982). Karyotypes in some pulse crops. Nucleus.

[75] Beauchamp C, Fridovich I (1971). Superoxide dismutase: Improved assays and an assay applicable to acrylamide gels. Anal. Biochem..

[76] Zou J, Yue J, Jiang W, Liu D (2012). Effects of cadmium stress on root tip cells and some physiological indexes in *Allium cepa* var *agrogarum* L.. Acta Biol. Cracov. Ser. Bot..

[77] Beers RF, Sizer IW (1952). Colorimetric method for estimation of catalase. J. Biol. Chem..

[78] Unyayar S, Celik A, Cekic FO, Gozel A (2006). Cadmium-induced genotoxicity, cytotoxicity and lipid peroxidation in *Allium sativum* and *Vicia faba*. Mutagenesis.

[79] Bates LS, Waldren RP, Teare ID (1973). Rapid determination of free proline for water stress studies. Plant Soil..

[80] Benedetti CE, Costa CL, Turcinelli SR, Arruda P (1998). Differential expression of a novel gene in response to coronatine, methyl jasmonate, and wounding in the Coi1 mutant of Arabidopsis. Plant Physiol..

[81] Al-Karaki GN (2001). Germination, sodium, and potassium concentrations of barley seeds as influenced by salinity. J. Plant Nutr..

[82] Ghoulam C, Fares K (2001). Effect of salinity on seed germination and early seedling growth of sugar beet (*Beta vulgaris* L.). Seed Sci. Technol..

[83] Cavusoglu D (2020). Role of β-carotene on alleviation of salt-induced stress in *Allium cepa* L.. Bulg. J. Crop Sci..

[84] Cavusoglu D (2020). Role of propolis in alleviation of detrimental effects of salt stress on some physiological and cytogenetical parameters in onion (*Allium cepa* L.). Bulg. J. Crop Sci..

[85] Cavusoglu K, Dogu F, Cavusoglu D (2019). Effects of sodium hypochlorite on some physiological and cytogenetical parameters in *Allium cepa* L. exposed to salt stress. Bangl. J. Bot..

[86] Cavusoglu K, Dincturk I, Cavusoglu D (2020). The effects of aspartic acid on some physiological and cytogenetical parameters in *Allium cepa* L. seeds germinated under salt stress. Bulg. J. Crop Sci..

[87] Cavusoglu K, Togay D, Cavusoglu D (2020). Physiological and cytogenetical effects of glutamine treatment in onion (*Allium cepa* L.) seeds exposed to salt stress. Bulg. J. Crop Sci..

[88] Ali RM (2000). Role of putrescine in salt tolerance of *Atropa belladonna* plant. Plant Sci..

[89] Kang D (2005). Jasmonic acid differentially affects growth, ion uptake and abscisic acid concentration in salt-tolerant and salt-sensitive rice cultivars. J. Agron. Crop Sci..

[90] Nishiyama R (2012). Transcriptome analyses of a salt-tolerant cytokinin-deficient mutant reveal differential regulation of salt stress response by cytokinin deficiency. PLoS One.

[91] Colebrook EH, Thomas SG, Phillips AL, Hedden P (2014). The role of gibberellin signalling in plant responses to abiotic stress. J. Exp. Biol..

[92] Cavusoglu K, Cadil S, Cavusoglu D (2017). Role of potassium nitrate (KNO_3_) in alleviation of detrimental effects of salt stress on some physiological and cytogenetical parameters in *Allium cepa* L.. Cytologia.

[93] Cavusoglu D, Cavusoglu K, Tabur S (2018). The effects of black cumin (*Nigella sativa* L.) seed extract on the seed germination, seedling growth, mitotic activity and chromosomal aberrations of *Allium cepa* L. under saline condition. ARPN J. Agric. Biol. Sci..

[94] Fernandes TCC, Mazzeo DEC, Marin Morales MA (2007). Mechanism of micronuclei formation in polyploidizated cells of *A. cepa* exposed to trifluralin herbicide. Pest. Biochem. Physiol..

[95] Radic S, Prolic M, Pavlica M, Pevalek-Kozlina B (2005). Cytogenetic effects of osmotic stress on the root meristem cells of *Centaurea ragusina* L.. Environ. Exp. Bot..

[96] Tabur S, Demir K (2009). Cytogenetic response of 24-epibrassinolide on the root meristem cells of barley seeds under salinity. Plant Growth Regul..

[97] Tabur S, Demir K (2010). Role of some growth regulators on cytogenetic activity of barley under salt stress. Plant Growth Regul..

[98] Tabur S, Yurtlu MD, Özmen S (2019). Role of humic acid against salt-induced cytotoxicity in *Hordeum vulgare* L.. Caryologia.

[99] Ozmen S, Tabur S (2020). Functions of folic acid (vitamin B9) against cytotoxic effects of salt stress in *Hordeum vulgare* L.. Pak. J. Bot..

[100] Fenech M (2000). The in vitro micronucleus technique. Mutat. Res..

[101] Norppa H, Falck GC (2003). What do human micronuclei contain?. Mutagenesis.

[102] Fenech M, Crott JW (2002). Micronuclei, nucleoplasmic bridges and nuclear buds induced in folic acid deficient human lymphocytes-evidence for breakage-fusion-bridge cycles in the cytokinesis block micronucleus assay. Mutat. Res..

[103] Ruan C, Lian Y, Lium J (1992). Application of micronucleus test in *Vicia faba* in the rapid deletion of mutagenic environmental pollutants. Chin. J. Environ. Sci..

[104] Sutan NA, Popescu A, Mihaescu C, Soare LC, Marinescu MV (2014). Evaluation of cytotoxic and genotoxic potential of the fungicide Ridomil in *Allium cepa* L.. Anal. Stiint Univ. Al I Cuza Iasi..

[105] Turkoglu S (2007). Genotoxicity of five food preservatives tested on root tips of *Allium cepa* L.. Mutat. Res..

[106] Leme DM, Marin-Morales MA (2009). *Allium cepa* test in environmental monitoring: A review on its application. Mutat. Res..

[107] El-Ghamery AA, El-Kholy MA, Abou El-Yousser MA (2003). Evaluation of cytological effects of Zn2+ in relation to germination and root growth of *Nigella sativa* L. and *Triticum aestivum* L.. Mutat. Res..

[108] Kalcheva VP, Dragoeva AP, Kalchev KN, Enchev DD (2009). Cytotoxic and genotoxic effects of Br-containing oxaphosphole on *Allium cepa* L. root tip cells and mouse bone marrow cells. Genet. Mol. Biol..

[109] Vranova E, Inze D, Van Breusegen F (2002). Signal transduction during oxidative stress. J. Exp. Bot..

[110] Srivastava AK, Singh D (2020). Assessment of malathion toxicity on cytophysiological activity, DNA damage and antioxidant enzymes in root of *Allium cepa* model. Sci. Rep..

[111] Ashraf M (2009). Biotechnological approach of improving plant salt tolerance using antioxidants as markers. Biotechnol. Adv..

[112] Kachout SS, Hamza KJ, Bouraoui NK, Leclerc JC, Ouerghi Z (2013). Salt-induced changes in antioxidative enzyme activities in shoot tissues of two atriplex varieties. Not. Bot. Hortic. Agrobot. Cluj-Napoca.

[113] Apel K, Hirt H (2004). Reactive oxygen species: Metabolism, oxidative stress, and signal transduction. Ann. Rev. Plant Biol..

[114] Celik O, Atak C (2012). The effect of salt stress on antioxidative enzymes and proline content of two Turkish tobacco varieties. Turk. J. Biol..

[115] Boughalleb F, Abdellaoui R, Mahmoudi M, Bakhshandeh E (2020). Changes in phenolic profile, soluble sugar, proline, and antioxidant enzyme activities of *Polygonum equisetiforme* in response to salinity. Turk. J. Bot..

[116] Boughalleb F, Abdellaoui R, Nbiba N, Mahmoudi M, Neffati M (2017). Effect of NaCl stress on physiological, antioxidant enzymes and anatomical responses of *Astragalus gombiformis*. Biologia.

[117] Haddadi BS, Hassanpour H, Niknam V (2016). Effect of salinity and waterlogging on growth, anatomical and antioxidative responses in *Mentha aquatica* L.. Acta Physiol. Plant..

[118] Merati MJ, Hassanpour H, Niknam V, Mirmasoumi M (2014). Exogenous application of penconazole regulates plant growth and antioxidative responses in salt-stressed *Mentha pulegium* L.. Plant Interact..

[119] Hossain Z, Lopez-Climent MF, Arbona V, Perez-Clemente RM, Gomez-Cadenas A (2009). Modulation of the antioxidant system in citrus under waterlogging and subsequent drainage. Plant Physiol..

[120] Asada K (1999). The water-water cycle in chloroplasts: Scavenging of active oxygens and dissipation of excess photons. Annu. Rev. Plant Physiol. Plant Mol. Biol..

[121] Sytar O (2013). Heavy metal-induced oxidative damage, defense reactions, and detoxification mechanisms in plants. Acta Physiol. Plant..

[122] Scandalios JG (1993). Oxygen stress and superoxide dismutases. Plant Physiol..

[123] Chelikani P, Fita I, Loewen PC (2004). Diversity of structures and properties among catalases. Cell Mol. Life Sci..

[124] Esfandiari E, Shekari F, Shekari F, Esfandiari M (2007). The effect of salt stress on antioxidant enzymes’activity and lipid peroxidation on the wheat seedling. Not. Bot. Hortic. Agrobot. Cluj-Napoca.

[125] Demiral T, Turkan I (2004). Does exogenous glycinebetaine affect antioxidative system of rice seedlings under NaCl treatment?. J. Plant Physiol..

[126] Farmer EE, Mueller MJ (2013). ROS-mediated lipid peroxidation and RES-activated signaling. Annu. Rev. Plant Biol..

[127] Lima ALS, DaMatta FM, Pinheiro HA, Totola MR, Loureiro ME (2002). Photochemical responses and oxidative stress in two clones of *Coffea canephora* under water deficit conditions. Environ. Exp. Bot..

[128] Abdelaal KA (2020). Treatment of sweet pepper with stress tolerance-inducing compounds alleviates salinity stress oxidative damage by mediating the physio-biochemical activities and antioxidant systems. Agronomics.

[129] Ahanger MA, Mir RA, Alyemeni MN, Ahmad P (2020). Combined effects of brassinosteroid and kinetin mitigates salinity stress in tomato through the modulation of antioxidant and osmolyte metabolism. Plant Physiol. Biochem..

[130] Ahmad P (2019). Silicon (Si) supplementation alleviates NaCl toxicity in mung bean [*Vigna radiata* (L.)Wilczek] through the modifications of physio-biochemical attributes and key antioxidant enzymes. J. Plant Growth Regul..

[131] Karray-Bouraoui N (2010). Enzymatic and non-enzymatic antioxidant responses of two *Mentha pulegium* provenances to salt stress. Med. Plant Res..

[132] Ashraf M, Foolad MR (2007). Roles of glycine betaine and proline in improving plant abiotic stress resistance. Environ. Exp. Bot..

[133] Sadiqov ST, Akbulut M, Ehmedov V (2002). Role of Ca^2+^ in drought stress signaling in wheat seedlings. Biochemistry.

[134] Munns R, Tester M (2008). Mechanisms of salinity tolerance. Annu. Rev. Plant Biol..

[135] Kumar SG, Reddy AM, Sudhakar C (2003). NaCl effects on proline metabolism in two high yielding genotypes of mulberry (*Morus alba* L.) with contrasting salt tolerance. Plant Sci..

[136] Martinez CA, Maestri M, Lani EG (2003). In vitro salt tolerance and proline accumulation in Andean potato (*Solanum* spp.) differing in frost resistance. Plant Sci..

[137] Wang XS, Han JG (2009). Changes in proline content, activity, and active isoforms of antioxidative enzymes in two alfalfa cultivars under salt stress. Agric. Sci. China..

[138] Misra N, Gupta AK (2005). Effect of salt stress on proline metabolism in two high yielding genotypes of green gram. Plant Sci..

[139] Jaleel CA (2007). Studies on germination, seedling vigour, lipid peroxidation, and proline metabolism in *Catharanthus roseus* seedlings under salt stress. S. Afr. J. Bot..

[140] Yazici I, Turkan I, Sekmen AH, Demiral T (2007). Salinity tolerance of purslane (*Portulaca oleracea* L.) is achieved by enhanced antioxidative system, lower level of lipid peroxidation, and proline accumulation. Environ. Exp. Bot..

[141] Aly AA, Latif HH (2011). Differential effects of paclobutrazol on water stress alleviation through electrolyte leakage, phytohormones, reduced glutathione and lipid peroxidation in some wheat genotypes (*Triticum aestivum* L.) grown in-vitro. Rom. Biotech. Lett..

[142] Lutts S, Majerus V, Kinet JM (1999). NaCl effects on proline metabolism in rice (*Oryza sativa*) seedlings. Physiol. Plant..

[143] Shannon MC, Grieve CM (1999). Tolerance of vegetable crops to salinity. Sci. Hortic..

[144] Matysik J, Alia-Bhalu B, Mohanty P (2002). Molecular mechanisms of quenching of reactive oxygen species by proline under stress in plants. Curr. Sci..

[145] Khedr AHA, Abbas MA, Wahid AAA, Quick WP, Abogadallah GM (2003). Proline induces the expression of salt-stress-responsive proteins and may improve the adaptation of *Pancratium maritimum* L. to salt-stress. Exp. Bot..

[146] Dauer WT, Worman HJ (2009). The nuclear envelope as a signaling node in development and disease. Dev. Cell..

[147] Baker AJM (1981). Accumulators and excluders-strategies in the response of plants to heavy metals. J. Plant Nutr..

